# Selection of IgG Variants with Increased FcRn Binding Using Random and Directed Mutagenesis: Impact on Effector Functions

**DOI:** 10.3389/fimmu.2015.00039

**Published:** 2015-02-04

**Authors:** Céline Monnet, Sylvie Jorieux, Rémi Urbain, Nathalie Fournier, Khalil Bouayadi, Christophe De Romeuf, Christian K. Behrens, Alexandre Fontayne, Philippe Mondon

**Affiliations:** ^1^LFB Biotechnologies, Lille, France; ^2^LFB Biotechnologies, Courtaboeuf, France; ^3^MilleGen, Labège, France

**Keywords:** complement C1q, directed mutagenesis, Fc engineering, Fc gamma receptors, FcRn, IgG, random mutagenesis, therapeutic mAbs

## Abstract

Despite the reasonably long half-life of immunoglogulin G (IgGs), market pressure for higher patient convenience while conserving efficacy continues to drive IgG half-life improvement. IgG half-life is dependent on the neonatal Fc receptor (FcRn), which among other functions, protects IgG from catabolism. FcRn binds the Fc domain of IgG at an acidic pH ensuring that endocytosed IgG will not be degraded in lysosomal compartments and will then be released into the bloodstream. Consistent with this mechanism of action, several Fc-engineered IgG with increased FcRn affinity and conserved pH dependency were designed and resulted in longer half-life *in vivo* in human FcRn-transgenic mice (hFcRn), cynomolgus monkeys, and recently in healthy humans. These IgG variants were usually obtained by *in silico* approaches or directed mutagenesis in the FcRn-binding site. Using random mutagenesis, combined with a pH-dependent phage display selection process, we isolated IgG variants with improved FcRn-binding, which exhibited longer *in vivo* half-life in hFcRn mice. Interestingly, many mutations enhancing Fc/FcRn interaction were located at a distance from the FcRn-binding site validating our random molecular approach. Directed mutagenesis was then applied to generate new variants to further characterize our IgG variants and the effect of the mutations selected. Since these mutations are distributed over the whole Fc sequence, binding to other Fc effectors, such as complement C1q and FcγRs, was dramatically modified, even by mutations distant from these effectors’ binding sites. Hence, we obtained numerous IgG variants with increased FcRn-binding and different binding patterns to other Fc effectors, including variants without any effector function, providing distinct “fit-for-purpose” Fc molecules. We therefore provide evidence that half-life and effector functions should be optimized simultaneously as mutations can have unexpected effects on all Fc receptors that are critical for IgG therapeutic efficacy.

## Introduction

Therapeutic monoclonal antibodies (mAbs) have proven successful in the clinic and are now in widespread use for the treatment of a variety of diseases including cancer, autoimmune, and infectious diseases. At the moment, new therapeutic mAbs are undergoing clinical trials at a record pace constantly filling very dynamic business pipelines (1). Most therapeutic mAbs are human or humanized molecules of the immunoglogulin G isotype (IgG), specifically IgG1, IgG2, and IgG4, which bind with high affinity the human neonatal Fc receptor (FcRn), in a strictly pH-dependent manner. This Fc/FcRn binding is largely responsible for the long half-life of IgGs as FcRn prevents IgG intracellular degradation. Following pinocytosis, IgGs are internalized into the endosomes of cells where the low pH (pH 5–6) promotes binding to FcRn with nanomolar affinity. Bound IgG-Fc/FcRn complexes are recycled back to the cell surface and dissociate at the neutral pH of the extracellular fluid, returning thus to the blood circulation. Due to the same mechanism, FcRn is also involved in IgG transport across placental, fetomaternal, and polarized cellular barriers (2, 3). This property has been successfully exploited for pulmonary delivery and intranasal immunization with Fc-fusion proteins (4, 5). Recently, FcRn was shown to enable transepithelial transport of Fc-targeted nanoparticles delivered orally in mice. This innovative study paves the way for potential oral administration to treat chronic diseases (6). FcRn properties in transport and catabolism mainly occur in endothelial and epithelial cells. In human, FcRn is also expressed by many immune cells such as macrophages, monocytes, and dendritic cells. Several recent studies demonstrated unexpected roles for FcRn in antigen presentation (7–9) and phagocytosis (10) revealing a wide range of functions for this receptor in immunological and non-immunological mechanisms. A fundamental understanding of FcRn’s versatile roles will be essential to guide the development of therapeutic mAbs- and Fc-fusion proteins (11), especially when engineering Fc variants with modulated FcRn binding (12).

Given the considerable role of FcRn in antibody homeostasis, the IgG–Fc/FcRn interaction has been extensively studied for the last 20 years by directed mutagenesis (13) and crystallography (14). The FcRn-binding site is structurally conserved among species and is localized in the CH2/CH3 interdomain Fc region. Based on this knowledge, Fc-engineered mAbs with increased FcRn affinity and conserved pH dependency were obtained by alanine scanning (13), directed mutagenesis (15), and *in silico* approach (16–18) focused on the FcRn-binding site. The resulting Fc variants usually include two to three substitutions in or close to the FcRn-binding site. Consistent with FcRn’s role in IgG catabolism, such engineered mAbs showed longer half-life *in vivo* in human FcRn-transgenic mice (hFcRn mice) and/or cynomolgus monkeys (16–24). For instance, Fc variants of the anti-VEGF bevacizumab and the anti-EGFR cetuximab (Fc-LS, M428L/N434S) demonstrated half-lives extended up to almost fivefold in hFcRn mice and threefold in cynomolgus monkeys (18). When introduced in a CTLA4-Fc-fusion protein, this Fc-LS variant exhibited increased half-life in cynomolgus monkeys as well, albeit at a lower level than for mAbs (1.4-fold increase) (25). Remarkably, for the first time in healthy humans an Fc-engineered anti-RSV mAb (motavizumab-YTE, M252Y/S254T/T256E) showed a significant serum half-life extension (up to 100 days, two- to fourfold increase), fully confirming in humans the results previously obtained in animal models (19, 26). This proof of concept validated for the first time in humans the usefulness of increasing FcRn binding to obtain mAbs with extended half-life. Such improved therapeutic mAbs should prove attractive for developers as they offer several advantages allowing a positive differentiation from the competition. First, treatment intervals could be increased while keeping the same dosing, resulting in better patient convenience and reduced costs. Second, for the same efficacy, with the same dosing interval, drug quantities may be lowered, reducing costs as well. Finally, the use of the same dose and dosing interval as the parent antibody should result in higher drug exposure. Interestingly, when this setting was used, greater anti-tumor activity was observed for the Fc-LS variants tested in hFcRn/Rag1^−/−^ tumor xenografted mice, showing a positive correlation between FcRn-binding enhancement and *in vivo* efficacy (18). Whether this improved therapeutic efficacy is solely due to prolonged drug exposure, or also due to other FcRn-mediated mechanisms, needs to be elucidated.

On the other hand, it is now clearly established that the therapeutic efficacy of mAbs substantially rely on their ability to engage the immune system via their Fc domain (27). This Fc-dependent engagement is mainly mediated by crosslinking of Fcγ receptors (FcγRs) widely expressed on many innate immune effector cells such as NK cells, monocytes, macrophages, and neutrophils. Immune effector cells express one or more activating FcγRs, FcγRI (CD64), FcγRIIA (CD32a), and FcγRIIIA (CD16a), and/or the only inhibitory receptor FcγRIIB (CD32b). Following engagement of their activating FcγRs, these cells kill tumor or infected cells through antibody-dependent cellular cytotoxicity (ADCC) or phagocytosis (ADCP). Natural polymorphisms of these FcγRs have been described in humans, resulting in modulated affinities for IgG subclasses. These FcγRs polymorphisms, especially the FcγRIIIA polymorphism (V/F158), influence patients’ response to treatment with therapeutic mAbs (28). More favorable clinical responses were indeed observed in patients homozygous for the higher-affinity allele of FcγRIIIA (V158) in various disorders: with the anti-CD20 rituximab in non-Hodgkin lymphomas (29), in immune thrombocytopenia (30), and in rheumatoid arthritis (31), with the anti-HER2 trastuzumab in metastatic breast cancer (32), with the anti-EGFR cetuximab in metastatic colorectal cancer (33), and with the anti-TNFα infliximab in Crohn’s disease (34). These findings suggest a crucial role for FcγRIIIA in the *in vivo* activity of therapeutic mAbs because the V158 polymorphic variant displays a higher affinity for IgG1 and increased ADCC. Therefore, in order to enhance therapeutic activity, many protein-Fc-engineered mAbs with increased FcγRIIIA binding were designed (35) using alanine scanning (13, 36), random mutagenesis (37), and *in silico* approach (38). Glyco-engineering was also successfully used to that aim, as Fc glycan content strongly influences Fc/FcγRIIIA interaction, contrarily to the Fc/FcRn interaction, which is not dependent on the glycan structure. The absence of fucose residues on the Fc glycan moiety of mAbs has long been associated with increased affinity for FcγRIIIA (39). Afucosylated or low fucosylated mAbs with strongly enhanced cytotoxicity were obtained by cellular engineering. More than 20 glyco-engineered mAbs, with higher ADCC, are now in clinical studies and two have already been approved since 2012 (mogamulizumab and obinutuzumab) confirming the success of this approach (40).

In a previous study, we demonstrated that glyco-engineering to improve cytotoxicity and protein-Fc engineering to increase half-life can be combined to further optimize therapeutic mAbs (41). In our approach, we used a random mutagenesis technology with a pH-dependent phage display selection process to isolate several Fc variants of human IgG1 with improved FcRn affinity. Interestingly, some of the identified mutations, enhancing Fc/FcRn interaction, were located at a distance from the FcRn-binding site validating our random molecular approach. When produced as low fucosylated molecules (EMABling^®^ platform), our IgG variants demonstrated increased serum persistence in hFcRn mice (half-life of 23 h for the IgG-WT and 64 h for our best IgG variant) as well as conserved enhanced ADCC for five out of our six best variants. In this study, we used directed mutagenesis to further characterize the effect of the mutations previously selected on the Fc/FcRn interaction. Because these mutations are distributed on the whole Fc sequence, we assessed the binding of several Fc variants to the complement C1q and FcγRs, and evaluated effectors functions, ADCC and CDC, as well. We therefore obtained numerous IgG variants with increased FcRn binding and different binding patterns to other Fc receptors and C1q, including variants without any effector function, providing distinct “fit-for-purpose” Fc molecules.

## Materials and Methods

### Expression and Purification of Human FcRn

Soluble human FcRn was produced by GTP Technology using the baculovirus expression system as previously described (42). The α-chain cDNA encoding the leader peptide and extracellular domains (codons 1–290) was tagged with a TEV sequence and a 6× polyhistidine tag. The derivative α-chain and the β2-microglobulin chain were cloned into pFastBacDual under the P10 and polyhedrine promoters, respectively. A fusion protein containing the β2-microglobulin chain and the α-chain fused to the amino terminal part of the bacteriophage p3 protein and the CVDE protein (FcRn-p3) was also produced. Proteins with more than 90% purity were obtained after IgG-Sepharose and IMAC purification steps.

### Phage-ELISA Assays of Fc Variants

The human Fc gene encoding amino acid residues 226–447 [EU index as in Kabat (43) and Supplementary Material], derived from a human IgG1 heavy chain (G1m1, 17 allotype), was cloned into the phagemid vector pMG58 (pMG58-Fc226) as a *Bam*HI*/Eco*RI fragment using standard PCR protocols. Wild-type Fc (Fc-WT) and variants were expressed on the surface of the bacteriophage M13 (41, 44) and their FcRn-binding characteristics were determined using an ELISA test at pH 6.0. Briefly, *E. coli* XL1-Blue bacteria containing the Fc variants (in pMG58 vector) were grown as separate clones on a 96-well plate in 800 μl cultures in 2YT medium supplemented with 100 μg/ml ampicillin, 15 μg/ml tetracycline, and 1% (w/v) glucose at 30°C, 230 rpm until OD_600nm_ = 0.6 is reached. Cells were then infected with M13 helper phage (M13KO7, New England Biolabs, ratio bacteria/phage = 1/3) at 37°C for 20 min. Phage-Fc were produced overnight at 26°C, 230 rpm in 2YT/ampicillin/glucose with 0.5 mM IPTG and 30 μg/ml kanamycin and recovered in the supernatants after centrifugation for 30 min. at 3,000 × *g*. These supernatants were directly diluted (1/2 and 1/4) in phosphate buffer pH 6.0 (100 mM sodium phosphate, 50 mM sodium chloride pH 6.0, called P6), supplemented with 5% skimmed milk and 0.1%Tween-20, and tested on Maxisorp immunoplates previously coated with 0.25 μg FcRn-p3/well and blocked with 5% skimmed milk in P6. After incubation for 2 h at 37°C, wells were washed three times with P6/0.1% Tween-20 and bound phages were detected with an HRP anti-M13 antibody (GE Healthcare).

### Directed Mutagenesis

Directed mutagenesis was performed to construct positive controls and remove or add point mutations by overlap PCR using standard protocols. All the variants were constructed with overlapping primers containing the desired mutations and the primers MG-619 (5′-*AGTACTGACTCTACCTA*GGATCCTGCCCACCGTGC-3′) and MG-621 (5′-*ACTGCTCGATGTCCGTA*CTATGCGGCCGCGAATTC-3′) for cloning (*Bam*HI and *Eco*RI restriction sites are underlined and italic characters correspond to non-specific tails). All the variants constructed by directed mutagenesis are described in patent applications WO2010106180 (45) and WO2012175751 (46). The positive controls for FcRn were the double variant T250Q/M428L (16, 17) (Fc-QL), the triple variant M252Y/S254T/T256E (15) (Fc-YTE), and the double variant M428L/N434S (Fc-LS) (18). The positive control for FcγRIIIA was S239D/I332E (C1) (38), for the complement C1q; K326W/E333S (C3) (47), for FcγRIIA; G236A (C4) (48), and for FcγRIIB; S267E/L328F (C5) (49).

### Production of IgG Variants in YB2/0 Cells

The Fc WT and variants were produced in IgG format with an anti-CD20 specificity based on the Fv domain of CAT 13.6E12 (50), using the YB2/0 cell line (ATCC, CRL-1662). Heavy and light chains of the anti-CD20 antibody were cloned into the CHK622-08 vector, optimized for production in Y2B/0 cells, as previously described (41). Each linearized expression construct was introduced by electroporation into 5 × 10^6^ YB2/0 cells. Cells were then expanded to 25,000 cells/ml in RPMI 1640 medium + 5% v/v dialyzed FCS (Invitrogen) and dispensed in 1 ml/well into 24-well plates. After 3 days, selection pressure was applied by adding geneticin (Invitrogen) and methotrexate (Sigma) to obtain final concentrations of 0.5 g/l and 25 mM respectively, in 2 ml/well. After 11 days, resistant cells were pooled for each construct and progressively expanded with DMEM medium + 5% v/v Ultra-low IgG FCS (Invitrogen) until two 2 l-roller bottles, each containing 0.9 l of cell suspension can be incubated at 2 rpm. Cells were allowed to grow and die (4–5 days) before supernatant collection, clarification by low-speed centrifugation and volume reduction by ultra-filtration using a Pellicon XL Filter (Millipore). The concentrated culture supernatants were injected into a HiTrap protein A FF column (GE Healthcare). Bound antibodies were eluted with 0.1 M sodium citrate, pH 3.0, and fractions were neutralized using 100 μl of 1 M Tris–HCl pH 7.5/ml of elution buffer. Fractions containing the antibodies were pooled and dialyzed against PBS pH 6.0, and the samples were sterile-filtered (0.22 μm) and stored at 4°C. The purified IgGs were analyzed using SDS–PAGE under non-reducing and reducing conditions as well as analytical gel filtration (on Superdex 200 10/300 GL with an AKTA prime system, GE Healthcare) in order to estimate aggregate contents and potential contaminants. Coomassie Blue-stained gels indicated that the IgGs, whatever the mutations, had >95% purity and displayed the characteristic heavy and light chain bands. Purified IgGs were considered suitable for biological tests when aggregate rates were below 5%. Limulus Amebocyte Lysate (LAL) endotoxin test Gel Clot method was further used to test purified IgGs for the presence of endotoxins. Endotoxin levels of the purified IgGs were below 7 UI/mg.

### FcRn Binding of IgG Variants by Surface Plasmon Resonance

The interaction of IgG variants produced in Y2B/0 with immobilized recombinant human FcRn was monitored by surface plasmon resonance (SPR) detection on a BIAcore X100 instrument using a CM5 sensor chip (BIAcore, GE Healthcare), as previously described (41). All measurements were performed at 25°C with IgG concentrations ranging from 1 to 200 nM at a flow rate of 10 μl/min. IgGs were diluted in PBS/Tween-20 (50 mM sodium phosphate, pH 6.0, 150 mM NaCl, 0.02% NaN_3_, 0.01% Tween-20), which is used as running buffer in equilibrium binding experiments. Data were collected for 10 min and a 1 min pulse of PBS, pH 8.0 containing 0.05% Tween-20 was used to regenerate surfaces. The equilibrium RU observed for each injection was plotted against the IgG concentration. The equilibrium *K*_D_ values were determined using the steady-state affinity model included in the BIA evaluation software version 3.1.

### ADCC Activity

Human natural killer cells (NK cells) were purified from the peripheral blood of healthy volunteer donors by the negative depletion technique developed by Miltenyi. The ADCC test comprises incubating the NK cells with CD20-expressing Raji target cells, in the presence of different concentrations of anti-CD20 antibodies. After 16 h of incubation, the cytotoxicity induced by the anti-CD20 antibodies was measured by quantifying in cell supernatants the level of the intracellular enzyme lactate dehydrogenase (LDH) released from lysed target cells. The specific lysis results are expressed as the percentage of lysis as a function of antibody concentration. EC_50_ values (antibody concentration inducing 50% of maximum lysis induced by the IgG-WT) and Emax (percentage of maximum lysis) were calculated using the software GraphPad PRISM.

### CDC Activity

The CD20-expressing Raji target cells were incubated with different concentrations of anti-CD20 antibodies (0–5,000 ng/ml) in the presence of baby rabbit serum as a source of complement (Cedarlane, dilution to 1/10). After 1 h of incubation at 37°C, the level of LDH released in the supernatant by the lysed target cells was measured chromogenically (Roche Applied Sciences Cytotoxicity Detection Kit) and used to quantify the complement-dependent cytotoxicity mediated by the antibodies. The results were expressed as a percentage of lysis. EC_50_ (quantity of antibody that induces 50% of maximum lysis) and *E*_max_ (percentage of maximum lysis) were calculated using the software GraphPad PRISM.

### Production of IgG Variants in HEK293-F Cells

The Fc WT and variants were cloned into the eukaryotic expression vector pMGM05-CD20 as a *Bam*HI*/Not*I fragment using standard PCR protocols. The pMGM05-CD20 vector is derived from pCEP4 (Invitrogen) and contains the heavy chain of the anti-CD20 antibody previously expressed in Y2B/0 cells [based on the Fv domain of CAT 13.6E12 (50)]. The light chain of this antibody was inserted into a similar vector derived from pCEP4 (pMGM01-CD20). FreeStyle™ HEK293-F cells (Invitrogen), cultured in 24-well plates, were co-transfected with pMGM01-CD20 and pMGM05-CD20 vectors (Fc-WT and variants) in equimolar amounts (250 ng/ml) with FreeStyle™ MAX reagent (1 μl/ml) using standard protocols (Invitrogen). Cells were cultured in suspension in serum-free medium for 7 days post-transfection and supernatants (1 ml) containing IgG were harvested after centrifugation of the cells at 100 g for 10 min. IgG secreted in the supernatants were quantified using an ELISA assay on recombinant protein L (Pierce), with purified anti-CD20 antibody produced in HEK293-F cells used as standard. Supernatants and standard antibody, serially diluted in PBS/0.05% Tween-20, were tested on Maxisorp immunoplates (Nunc) previously coated with 0.25 μg protein L/well and blocked with 5% skimmed milk in PBS. After incubation for 1 h at 37°C, wells were washed three times with PBS/0.05% Tween-20. Bound IgG variants were detected with an HRP goat anti-human IgG (γ chain specific) F(ab′)_2_ fragment (Sigma). IgG variants produced were quantified (1–4 μg/ml) using the standard curve.

### ELISA Tests of IgG Variants Produced in the Supernatants of HEK293-F Cells

The IgG variants were tested for their binding to the human complement C1q and several human Fc receptors by ELISA: C1q complement (Calbiochem), FcγRIIIA-V158 (R&D system), FcRn-p3, FcγRIIA-R131 (R&D system), and FcγRIIB (R&D system). ELISA tests were performed in PBS for all effector molecules except for FcRn, which was realized in P6. Maxisorp immunoplates were coated with 0.5 μg C1q complement/well in PBS, 0.1 μg FcγRIIIA-V158/well in PBS, or 0.1 μg FcRn-p3/well in P6. Immobilizer nickel chelate plates (Nunc) were coated with 0.1 μg FcγRIIA-R131/well or 0.4 μg FcγRIIB/well in 0.01 M KCl. After coating overnight at 4°C, plates were washed two times with PBS/0.05% Tween-20 (or P6/0.05% Tween-20 for FcRn) and saturated with PBS/4% BSA (or P6/4% BSA for FcRn) for 2 h at 37°C. In parallel, supernatants were diluted in PBS to a final IgG concentration of 0.5 μg/ml (or diluted in P6 to 0.3 μg/ml for the FcRn-binding test) and mixed with HRP F(ab′)_2_ goat anti-human F(ab′)_2_ at the same concentration for 2 h at room temperature. F(ab′)_2_-aggregated IgGs were then incubated under gentle agitation for 1 h at 30°C on the saturated ELISA plates without dilution for C1q, FcγRIIA-R131, and FcγRIIB (i.e., IgGs at 0.5 μg/ml), diluted in PBS to 0.25 μg/ml for FcγRIIIA-V158 or diluted in P6 to 0.0375 μg/ml for FcRn-p3. Plates were then revealed with TMB (Pierce) and absorbance read at 450 nm.

## Results

### Random Mutagenesis to Increase FcRn Binding

#### Use of MutaGen™ and Phage Display Technologies

The human IgG1 Fc gene encoding amino acid residues 226–447 (referred to as Fc-WT, for Fc Wild Type) was cloned into our modified phagemid vector (51). This Fc-WT molecule comprises the five most C-terminal amino acids of the hinge region and the entire CH2 and CH3 domains. In our previous study (41), several fully randomized libraries were generated using low fidelity human DNA polymerases to introduce random mutations homogeneously throughout the entire gene without any hot spot (MutaGen™ technology). Fc variants with improved FcRn binding were then isolated from these Fc-libraries using a pH-dependent phage display selection on human recombinant FcRn. Briefly, FcRn binding and washing steps were performed at pH 6.0 whereas bound Fc-phages were eluted at pH 7.4 to preserve Fc/FcRn interaction pH dependency, which is crucial for its physiological role. Two successive rounds of mutagenesis and selection (called MS1 and MS2) were performed to cumulate Fc mutations with a positive impact on Fc/FcRn interaction. Thus, for the MS2 step, the DNA template used to construct random libraries was a DNA pool of 42 variants with improved FcRn binding by phage-ELISA selected during the MS1 step.

Throughout MS1 and MS2 rounds, 500 Fc variants were isolated and ranked according to their FcRn-binding properties using a comparative phage-ELISA assay at pH 6.0. To set up this experiment, the double variant T250Q/M428L (Fc-QL) (17) and the triple mutant M252Y/S254T/T256E (Fc-YTE) (15) were constructed as positive controls. In this phage-ELISA assay (at pH 6.0) on human recombinant FcRn, the Fc-QL, and Fc-YTE control variants, expressed on the M13 bacteriophage, had a specific signal threefold stronger than the Fc-WT (average ratio of 3.2 for Fc-QL and 3.5 for Fc-YTE). As expected, none of the control variants Fc-QL and Fc-YTE nor the Fc-WT showed any binding at pH 7.4 (data not shown). During the MS1 round, a total of 227 different Fc variants were isolated, 139 were considered positive with a ratio/Fc-WT >2 and 73 were better than the positive control Fc-QL (ratio/Fc-WT >3.2). The best clone, S5A-41, had a ratio/Fc-WT of 9.0. During the MS2 round, 223 different mutated clones were isolated. Since the difference between the signals of the Fc-WT and the Fc variants was too great to be directly compared, these variants were ranked using a comparative phage-ELISA assay against the Fc-QL variant as reference. The best Fc variant isolated during MS1, S5A-41, was used as positive control on each phage-ELISA plate, its ratio/Fc-QL was 3.9 (±0.6). Among the 223 Fc variants tested, 209 Fc variants were better than the Fc-QL and 39 Fc variants were better than the S5A-41 variant. Sequences and phage-ELISA results obtained for the 20 best Fc variants from MS2 in comparison with the S5A-41 variant are detailed in the upper part of Table 1. To compare the variants from MS2 with the variants from MS1, an estimated ratio/Fc-WT was calculated by multiplying the ratio/Fc-QL of the variants with the ratio/Fc-WT of the Fc-QL (=3.2 ± 0.3) determined during MS1 (ratio/Fc-WT = 3.2 × ratio/Fc-QL). Using this calculation, the S5A-41 variant had a ratio/Fc-WT of 12.5 instead of 9.0 when tested directly in comparison with the Fc-WT during the MS1. Ranking of significant Fc variants from MS1 and MS2 using this calculation is illustrated in Table 2.

**Table 1:** **Characterization of Fc variants isolated during the MS2 process**.

Fc variants	Mutations	Ratio/Fc-QL	SD
**C6A-69**	**T307A/N315D/A330V/E382V/N389T/N434Y**	**8.9**	**1.7**
**C6A-78**	**T256N/A378V7S383N/N434Y**	**8.7**	**1.9**
**T5A-74**	**N315D/A330V/N361D/A378V/N434Y**	**8.6**	**1.6**
**C6A-74**	**V259I/N315D/N434Y**	**8.5**	**1.5**
**C6A-60**	**P230S/N315D/M428L/N434Y**	**8.4**	**1.8**
T5A-58	F241L/V264E/T307P/A378V/H433R	8.1	1.5
C6A-72	T250A/N389K/N434Y	8.0	1.1
T5A-93	V305A/N315D/A330V/P395A/N434Y	8.0	1.6
T5A-78	V264E/Q386R/P396L/N434S/K439R	8.0	1.5
T5A-87	N315D/A330V/O362R/N434Y	7.8	1.4
**C6A-66**	**E294del/T307P/N434Y**	**7.7**	**0.9**
C6A-85	V305A/N315D/A330V/N389K/N434Y	7.4	1.5
C8A-15	N315D/A327V/A330VA/397M/N434Y	7.4	1.8
T5A-89	P230T/F241L/V264E/D265G/A378V/N421T	7.1	1.2
T7A-92	V264E/P396L/S415N/N434S	6.7	1.5
T6A-57	P227L/V264E/A378V/N434S	6.4	1.7
T5A-94	V264E/A378T/P396L	5.8	1.0
T6A-75	P230T/N315D/Q362R/S426T/N434Y	5.7	1.3
C3A-13	C226G/N315D/A330V/N434Y	5.6	0.9
T5A-55	P230L/F241L/F243L/V264E/T307P/A378V	5.6	1.2
**S5A-41**	**P230T/V303A/K322R/N389T/F404L/N434S**	**3.9**	**0.6**
T6A-21	N315D/A378V/N434Y	3.1	0.5
T4A-04	N315D/A330V/E382V/N434Y	3.0	0.1
C3A-01	N315D/A330V/N434Y	2.5	0.1
C3A-03	N315D/N434Y	2.0	0.4
C6A-05	T307A/N315D/A330V/N434Y	1.9	0.4
M3A-01	N361D/N434Y	1.4	0.1

*Mutations and phage-ELISA ratios obtained for the 20 best Fc variants of MS2 compared with the positive control Fc-QL (upper part of the Table). The six variants chosen to be further characterized are in bold. Below the S5A-41 variant are listed the Fc variants that were characterized during MS2 and are combinations of two to four mutations also found in our six preferred variants (lower part of the table)*.

**Table 2:** **Comparison of Fc variants selected during MS1 and MS2**.

Fc variants	Mutations	Ratio/Fc-WT	Variant origin
**C6A-69**	**T307A/N315D/A330V/E382V/N389T/N434Y**	**28.4**	**MS2**
**C6A-78**	**T256N/A378V/S383N/N434Y**	**27.8**	**MS2**
C6A-78A	T256N/A378V/N434Y	27.8	MS2 + mutagenesis
**T5A-74**	**N315D/A330V/N361D/A378V/N434Y**	**27.6**	**MS2**
**C6A-74**	**V259I/N315D/N434Y**	**27.2**	**MS2**
**C6A-60**	**P230S/N315D/M428L/N434Y**	**26.8**	**MS2**
**C6A-66**	**E294del/T307P/N434Y**	**24.6**	**MS2**
T6A-21	N315D/A378V/N434Y	10.0	MS2
T4A-04	N315D/A330V/E382V/N434Y	9.6	MS2
**S5A-41**	**P230T/V303A/K322R/N389T/**	**9.0**	**MS1**
	**F404L/N434S**	**(12.5)**	
C3A-01	N315D/A330V/N434Y	8.0	MS2
C3A-03	N315D/N434Y	6.4	MS2
C6A-05	T307A/N315D/A330V/N434Y	6.1	MS2
S4A-02	A378V/N434Y	4.6	MS1
M3A-01	N361D/N434Y	4.5	MS2
L6B-41	P230S/M428L	4.4	MS1
S4A-11	N389T/N434Y	3.7	MS1
S3A-35	N434Y	3.5	MS1
B4A-13	P228L	3.5	MS1
B3A-32	P228R	3.1	MS1
B5A-35	P230S	2.8	MS1
S3A-05	N434S	2.7	MS1
B4A-22A	A378V	2.6	MS1 + mutagenesis
B5A-25B	N315D	2.1	MS1 + mutagenesis
B5A-15	M428L	2.0	MS1
S3A-25A	V264E	1.9	MS1 + mutagenesis
L3A-01	T256N	1.8	MS1
B3A-17	T307A	1.3	MS1

*Mutations and phage-ELISA ratios obtained in comparison with the Fc-WT. Analysis of the single mutations and the different combinations of mutations allowed us to ascertain the impact of the mutations included in our six preferred variants*.

#### Data Mining Using Results from Random Mutagenesis and Phage-ELISA

Given the high number of positive clones isolated, a data base was constructed to store and analyze the DNA sequences and the phage-ELISA results obtained for the Fc variants isolated during MS1 and MS2 processes. Sequence analysis of the positive Fc variants (i.e., variants with a ratio/Fc-WT >2, corresponding to 139 variants from MS1 and 219 variants from MS2) identified the following 9 key positions, which are positions mutated in more than 10% of the improved Fc variants: P230, F241, V264, T307, N315, A330, A378, N389, and N434. Remarkably, the position N434 is mutated in almost 80% of the positive variants, whereas positions V264, N315, and A378 are mutated in more than 40% of the positive variants (41). These findings are clearly noticeable for the 20 best Fc variants listed in Table 1, which all comprise 2–3 key positions combined with 1–3 other mutations (4.45 mutated amino acids on average). From our Fc variants, we chose the five best variants (C6A-69, C6A-78, T5A-74, C6A-74, and C6A-60) as well as the C6A-66 variant for more thorough characterization (in bold in Tables 1 and 2). The latter was selected for its originality to contain an amino acid deletion (E294Del). Data mining from our data base showed that many Fc variants isolated from MS1 and MS2 were combinations of one to four mutations also found in our six selected variants (six Fc variants from MS2, lower part of Table 1 and Fc variants from MS1 and MS2 assembled in Table 2). This allowed us to verify by comparison if each mutation included in our six selected variants had a real positive effect on the FcRn binding. In case that no appropriate variant for comparison was available, new variants were generated by directed mutagenesis to reveal the impact of associated mutations. The resulting variants were named as the parental clone with a letter added at the end of the name (Table 2).

The C6A-60 variant comprises four mutations (P230S/N315D/M428L/N434Y) each of which is present as single mutations in the Fc variants B5A-35 (P230S), B5A-25B (N315D), B5A-15 (M428L), and S3A-35 (N434Y) and where they had a direct impact on FcRn binding, with ratios/Fc-WT ranging from 2 to 3.5 each. The C6A-60 variant is also a combination of the two double variants L6B-41 (P230S/M428L) and C3A-03 (N315D/N434Y), which both display additive effects of the single mutations (ratios/Fc-WT of 4.4 and 6.4, respectively). Conversely, the final combination of the 4 mutations, C6A-60 exhibits synergistic effects with a ratio/Fc-WT >25, instead of 10–15 in case of additive effects of the mutations. The C6A-74 variant comprises the double variant C3A-03 (N315D/N434Y) as well, with the addition of one mutation, V259I that was never seen in another Fc variant but has a great impact on Fc/FcRn binding, increasing the ratio/Fc-WT from 6.4 to more than 25. The T5A-74 variant is composed of five mutations, three of which have been tested as single mutations in the Fc variants B5A-25B (N315D), B4A-22A (A378V), and S3A-35 (N434Y) and have demonstrated a positive impact on Fc/FcRn interaction. The impact of the N361D mutation is confirmed by comparison of the variants M3A-01 (N361D/N434Y) and S3A-35 (N434Y). Likewise, the contribution of the A330V mutation is confirmed by comparison of the variants C3A-03 (N315D/N434Y) and C3A-01 (N315D/A330V/N434Y). The C6A-78 variant comprises four mutations, three of which exert a positive effect as shown in the variants L3A-01 (T256N), B4A-22A (A378V), and S3A-35 (N434Y). The last mutation, N383S, was not included in any other variant and its removal, C6A-78A variant, seems to have no effect on FcRn binding. This mutation was therefore identified as unnecessary to improve FcRn binding. This result was confirmed by SPR with purified IgG variants (Table 4) and, therefefore, C6A-78 variant was replaced by C6A-78A in the subsequent tests. Finally, the C6A-69 variant includes six mutations, three of which were shown to have a positive effect in the mutation variants B3A-17 (T307A), B5A-25B (N315D), and S3A-35 (N434Y). The impact of the A330V was described above for the T5A-74 variant. The comparison of the S4A-11 (N389T/N434Y) and S3A-35 (N434Y) variants suggests a modest contribution for the N389T mutation that needs to be confirmed. The impact of the E382V mutation is confirmed by comparison of the C3A-01 (N315D/A330V/N434Y) and T4A-04 (N315D/A330V/E382V/N434Y) variants.

Overall, these data permitted to confirm the direct contribution of most of the key positions identified previously by sequence analyses: P230, T307, N315, A330, A378, and N434. Moreover, the key position V264E, which is not found in our six best variants, was also tested as a single mutation, and a ratio/Fc-WT of 1.9 was obtained. Interestingly, these analyses also permitted the identification of a position in the hinge region, P228 that induces a significant FcRn binding increase when mutated to L or R (ratio/Fc-WT of 3.5 and 3.1, respectively). This position was not identified as a key position but displays the strongest FcRn binding increase as a single mutation, similarly to the crucial position N434, mutated to Y or S (ratio/Fc-WT of 3.5 and 2.7, respectively).

#### Distribution of the Identified Mutations on the Fc Sequence

**Figure 1:**
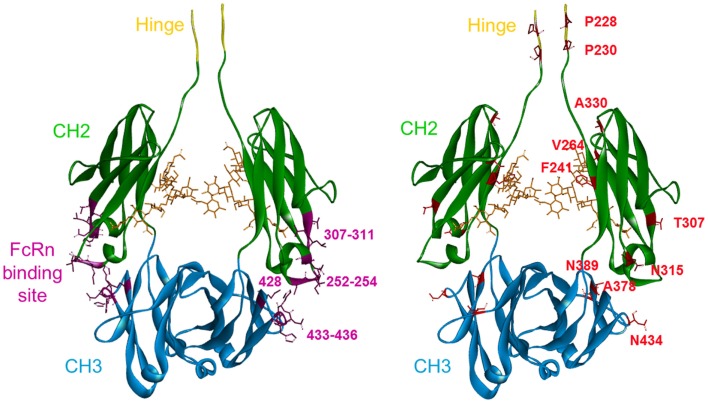
**3D representation of a model of the Fc fragment from the human IgG1 made with Discovery Studio software**. The last five amino acids of the hinge region are in yellow. The CH2 domain is in green and the CH3 domain is in blue. Left panel: in purple, the FcRn-binding site in the CH2–CH3 interdomain region, with the main positions used by others to increase FcRn binding highlighted: T250, M252, S254, T256, V259, T307, M428, H433, and N434. Right panel: the key positions identified in our study are highlighted in red. These mutations are distributed over the entire Fc sequence.

The amino acids previously identified were visualized on a 3D representation of the IgG1 Fc fragment modeled using crystallographic data (52) and Discovery Studio software (positions in red sticks, right panel of Figure 1). In parallel, the residues that have been involved in the Fc/FcRn interaction by crystallography (14) and directed mutagenesis (13, 53) were positioned on an analogous 3D representation. The FcRn-binding site encompasses three distinct zones in the Fc fragment at the interface between the CH2 and CH3 domains: residues 252–254, 307–311 in the CH2 domain, and residues 433–436 in the CH3 domain (positions in purple ribbon, left panel of Figure 1). Using this structural knowledge, several Fc variants with increased FcRn affinity have been obtained by alanine scanning (13), directed mutagenesis (15, 21), and *in silico* approaches (16–18) albeit focusing on the FcRn-binding site. These optimized Fc variants, developed by other groups, mainly include the residues T250, M252, S254, T256, V259, T307, V308, M428, H433, and N434 (positions in purple sticks, left panel of Figure 1), all located inside the FcRn-binding site. In contrast, the random mutagenesis method used to generate our Fc variants allowed to identify positions distributed over the whole Fc region. Among the positions identified, two are directly in the FcRn-binding site, one in the CH2 domain (T307), and the other in the CH3 domain (N434) and have been largely described previously (13, 18, 20). Three positions are close to the FcRn-binding site, one in the CH2 domain (N315), and the two others (A378 and N389) in the CH3 domain. Finally, five positions are located outside the FcRn-binding site: P228 and P230 in the lower hinge region and F241, V264, and A330 in the upper part of the CH2 domain. The mutations identified on these positions probably have a positive long-range effect on the overall structure of the Fc domain favoring the Fc/FcRn complex.

### Directed Mutagenesis to Increase FcRn Binding

Unexpectedly, the use of random mutagenesis and phage display selection revealed the contribution of mutations located in the Fc hinge domain on Fc/FcRn binding (P228R or L and P230S). Since these mutations were rarely used in our optimized variants, we combined them with our five best variants C6A-69, C6A-78A, T5A-74, C6A-74, and C6A-60 in an attempt to further increase their FcRn binding. New variants were constructed using directed mutagenesis by adding in the hinge region one or two mutations as follow: P230S, P228L, P228R, P228L/P230S, or P228R/P230S. These new variants were named based on the parental variant with a letter added at the end of the name (A–G) (Table 3). They were tested using phage-ELISA assay at pH 6.0 and result of each new variant was directly compared to its parental molecule to estimate the influence of the added mutation(s) (ratio new variant/parental variant). Depending on the parental variant, the results observed were very heterogeneous as the mutations displayed unpredictable and sometimes opposite effects. For instance, the mutation P228L could either have a negative (C6A-69 and C6A-60), a neutral (T5A-74), or a modest positive effect (C6A-78 and C6A-74). The mutations P230S, P228L/P230S, and P228R/P230S had mostly negative or neutral effects, with the exception of the combination of P228R/P230S with T5A-74 (twofold increase). Finally, the mutation P228R was the most efficient mutation with a neutral effect on two variants (C6A-69 and C6A-60) and a positive effect on three variants (C6A-78, T5A-74, and C6A-74), with ratios ranging from 2.2- to 3.3-fold increase compared to the parental molecule. These results exemplify the difficulty of designing optimized variants by directed mutagenesis, the resulting effect of the combination of several positive mutations being often unpredictable.

**Table 3:** **Comparison of Fc variants generated by directed mutagenesis to increase FcRn binding**.

Fc variants	Mutations	Ratio variant/parental variant	SD
C6A-69E	**P228R**/C6A-69	1.1	0.3
C6A-69	T307A/N315D/A330V/E382V/N389T/N434Y	1.0	0.2
C6A-69C	**P230S**/C6A-69	0.9	0.1
C6A-69D	**P228L**/C6A-69	0.5	0.1
C6A-78D	**P228R**/C6A-78A	3.3	0.8
C6A-78B	**P228L**/C6A-78A	1.7	0.2
C6A-78F	**P228R**/**P230S**/C6A-78A	1.1	0.2
C6A-78C	**P230S**/C6A-78A	1.1	0.3
C6A-78A	T256N/A378V/N434Y	1.0	0.1
C6A-78E	**P228L**/**P230S**/C6A-78A	0.7	0.0
T5A-74D	**P228R**/T5A-74	3.0	0.7
T5A-74F	**P228R**/**P230S**/T5A-74	2.0	0.6
T5A-74	N315D/A330V/N361D/A378V/N434Y	1.0	0.2
T5A-74C	**P228L**/T5A-74	0.9	0.2
T5A-74E	**P228L**/**P230S**/T5A-74	0.8	0.2
T5A-74B	**P230S**/T5A-74	0.7	0.2
C6A-74D	**P228R**/C6A-74	2.2	0.5
C6A-74C	**P228L**/C6A-74	1.3	0.3
C6A-74E	**P228L**/**P230S**/C6A-74	1.0	0.3
C6A-74	V259I/N315D/N434Y	1.0	0.2
C6A-74F	**P228R**/**P230S**/C6A-74	0.9	0.2
C6A-74A	**P230S**/C6A-74	0.7	0.1
C6A-60	**P230S**/N315D/M428L/N434Y	1.0	0.2
C6A-60D	**P228R**/C6A-60	0.9	0.2
C6A-60C	**P228L**/C6A-60	0.6	0.2

*The positions in the hinge domain (P228 and P230) identified by random mutagenesis were combined with our five best variants using directed mutagenesis and resulted in further increased FcRn binding in phage-ELISA assays*.

Successful combinations based on the parental variant C6A-78 were selected to be further characterized. IgG-WT and variants C6A-78, C6A-78A, C6A-78B, and C6A-78D were produced as full-length IgGs with the variable regions of the chimeric anti-human CD20 CAT 13.6E12 in Y2B/0 cells as described before (41). FcRn affinities of these IgGs were measured by SPR on immobilized recombinant human FcRn at pH 6.0 (Table 4). In this assay, the *K*_D_ values of the four IgG variants ranged from 19.8 to 33.1 nM as compared to 101.0 nM for the IgG-WT. This experiment confirmed that the C6A-78A variant has a similar affinity as C6A-78, albeit having one mutation less (N383S) and that the C6A-78B variant displays a slightly increased affinity. However, the positive impact of the mutation P228R (C6A-78D) was not confirmed. In the IgG molecule, this position P228 may be more constrained than in the phage display system, impeding the substitution by the arginine, whereas the substitution by the leucine seems to conserve a positive impact.

**Table 4:** **SPR affinity measures at steady state on immobilized recombinant human FcRn of IgG-WT and variants produced in YB2/0 cells**.

IgG variants	Mutations	KD (nM)	Ratio KD WT/variant
WT	None	101.0	1.0
C6A-78	T256N/A378V/S383/N434Y	33.1	3.1
C6A-78A	T256N/A378V/N434Y	26.1	3.9
C6A-78B	P228L/T256N/A378V/N434Y	19.8	5.1
C6A-78D	P228R/T256N/A378V/N434Y	25.3	4.0

### Impact of Mutations Increasing FcRn Binding on Effector Functions

#### IgG Variants Produced in Y2B/0 Cells

Together with the IgG-WT and the four IgG variants described above, IgG-YTE and the selected variants C6A-69, T5A-74, C6A-74, C6A-60, and C6A-66 were also produced as full-length IgGs with the variable regions of the chimeric anti-human CD20 CAT 13.6E12 (50). To combine the advantages of glyco- and protein-Fc engineering, these IgGs variants were produced in YB2/0 cells (EMABling^®^ platform), permitting low fucosylation of the IgG glycan moiety resulting in enhanced ADCC as previously described for two antibodies in clinical development, the anti-CD20 mAb ublituximab (54–56), and the anti-RhD mAb roledumab (57, 58). All such produced IgG variants conserved the same antigen binding properties as the IgG-WT and contain ~35% of fucosylated glyco-forms (data not shown). As described above, SPR measures on immobilized FcRn confirmed the increased FcRn affinity for all our IgG variants (41). IgGs were then tested for their capacity to elicit ADCC with purified NK cells against CD20-expressing target cells (Table 5, left column). As described before (41), our five best IgG variants displayed similarly high ADCC as the IgG-WT showing that they all have retained glyco-engineered improved ADCC activity. A slightly increased ADCC was even observed for the IgG variants C6A-74 and C6A-60 (x3.7 and x6.7, respectively). On the other hand, the C6A-66 variant, which contains the deletion of the amino acid E294, has completely lost ADCC activity. The C6A-78 and C6A-78A, differing from one mutation (N383S) that was confirmed to be dispensable for FcRn affinity, displayed similar ADCC. On contrary, the addition of mutations on position P228 seems to decrease ADCC, especially when arginine is used (C6A-78B and C6A-78D, x0.7 and 0.4, respectively, compared to a ratio of 1.1 for C6A-78). Interestingly, the IgG-YTE variant, despite being mutated exclusively in the FcRn-binding site, has lost its ADCC activity.

**Table 5:** **ADCC and CDC results of IgG-WT and IgG variants produced in YB2/0 cells**.

IgG variants	Mutations	ADCC: ratio EC50 WT/variant	CDC: ratio EC50 WT/variant
C6A-69	T307A/N315D/A330V/E382V/N389T/N434Y	2.6	< 0.1
C6A-78	T256N/A378V/S383N/N434Y	1.3	5.6
C6A-78A	T256N/A378V/N434Y	1.1	5.9
C6A-78B	P228L7T256N/A378V/N434Y	0.7	9.1
C6A-78D	P228R/T256N/A378V/N434Y	0.4	7.7
T5A-74	N315D/A330V/N361D/A378V/N434Y	1.8	< 0.1
C6A-74	V259l/N315D/N434Y	6.7	1.2
C6A-60	P230S/N315D/M428L/N434Y	3.7	5.6
C6A-66	E294Del/T307P/N434Y	<0.1	0.6
YTE	M252Y/S254T/T256E	0.2	<0.1

*Results are expressed as ratio EC_50_ WT/variant. Increased ratios therefore correspond to an improved ADCC or CDC activity of the IgG variant compared to the IgG-WT*.

*0–0.7; 0.7–2; 2–10*.

Immunoglobulins G were also tested for their ability to induce CDC activity against CD20-expressing target cells in presence of baby rabbit serum (Table 5, right column). The level of CDC activity varies between IgG variants. C6A-78, C6A-78A, C6A-78B, C6A-78D, and C6A-60 variants have a CDC activity significantly higher than that of IgG-WT whereas C6A-69, T5A-74, and C6A-66 displayed low CDC activity. The CDC activity of C6A-74 variant is similar to that of IgG-WT. Moreover, the IgG-YTE variant has also lost CDC activity.

Overall, these results show that the selected variants for increased FcRn binding have different ADCC and CDC activities. One variant, C6A-60, has increased ADCC and CDC and one variant, C6A-74, has increased ADCC and conserved CDC. Two variants, C6A-69 and T5A-74, have conserved ADCC and low CDC. Two variants, C6A-78 and C6A-78A, have conserved ADCC and increased CDC. Two variants, C6A-78B and C6A-78D, have decreased ADCC and increased CDC. Finally, one variant (C6A-66) has lost both ADCC and CDC activities, a feature also observed for the positive control IgG-YTE.

#### IgG Variants Produced in HEK Cells

Using the same antigen specificity as previously described, IgG-WT and variants were expressed in HEK293-F cells, a classical cell line, which produces IgGs with high fucose content (80–90%) resulting in low ADCC as compared to IgGs produced in YB2/0 cells. To rapidly compare numerous variants for their binding properties to several Fc effectors (C1q, FcγRIIIA-V158, FcγRIIA-R131, and FcγRIIB and FcRn), IgGs were produced in small quantities (1 ml), titrated in cell supernatants, and directly tested by ELISA. Results for each receptor were expressed as a ratio of specific signal obtained for the IgG variant compared to the signal of the IgG-WT (Table 6). Our six previously selected IgG variants were tested thereby, with eight new improved combinations based on the results obtained by phage-ELISA (Table 3). Several positive controls were produced as well to set up the experiments. For FcRn, we used the IgG-QL and IgG-YTE variants described above but also the double variant M428L/N434S (Fc-LS) (18). The positive control for FcγRIIIA was S239D/I332E (C1) (38), for the complement C1q; K326W/E333S (C3) (47), for FcγRIIA; G236A (C4) (48), and for FcγRIIB; S267E/L328F (C5) (49). Results for the positive controls C1–C5 are in agreement with published and patented data (US 2009/0042291 A1 and EP2386574 A2), validating our experimental setting. The C1 variant has increased binding for the three FcγRs tested. The C3 and C4 variants are selectively improved for one receptor each, the complement C1q and the FcγRIIA, respectively. The C5 variant binding is largely improved for FcγRIIB but also FcγRIIA-R131. As expected, the three positive controls IgG-LS, -QL, and -YTE display increased FcRn-binding ability (ratios 4.46, 2.39, and 2.06, respectively). All our improved IgG variants demonstrate similar increase (ratios between 2.83 and 4.72), with no significant differences. This ELISA assay was not discriminant enough to reveal potential slight differences between the variants for their FcRn binding. Interestingly, the IgG-YTE demonstrated a decreased binding capacity to the complement C1q and to the three FcγRs tested, in correlation with the functional results obtained with IgGs produced in YB2/0 cells (Table 5). Our variants display diverse binding patterns: selective decrease for FcγRIIIA (C6A-60), decrease for all the receptors (C6A-66, C6A-69, and C6A-69E), no change for any receptor (C6A-74, C6A-74C, and C6A-74D), slight increase for all the receptors (C6A-78, C6A-78A, C6A-78B, and C6A-78D), and decrease for the complement C1q binding (T5A-74, T5A-74D, and T5A-74F). The complement C1q binding results are in good correlation with the CDC results obtained with IgGs produced in YB2/0 cells. Conversely, FcγRIIIA binding results are significantly different from the ADCC results previously obtained (Table 5). For instance, C6A-60 and C6A-74, which displayed increased ADCC as low-fucose molecules, demonstrate decreased or conserved FcγRIIIA binding, respectively, when produced in HEK293 cells. Altogether, in accordance to what we observed for the IgG molecules produced in Y2B/0 cells, these results show that the variants selected for increased FcRn binding exhibit different binding patterns to the complement C1q and the FcγRs.

**Table 6:** **Binding cartography of IgG variants produced in HEK293 cells on the complement C1q, FcγRIIIA-V158, FcγRIIA-R131, FcγRIIB, and FcRn**.

IgG variants	Mutations	ELISA: ratio variant/WT									
	
		C1q	SD	FcγRlllaV	SD	FcγRllaR	SD	FcγRllb	SD	FcRn	SD
C1	S239D/I332E	0.80	0.20	7.85	3.04	5.77	1.55	5.69	3.15	1.02	0.12
C3	K326W/E333S	>30.00	ND	0.55	0.18	1.09	0.71	1.17	0.28	1.25	0.29
C4	G236A	0.68	0.14	0.65	0.17	9.06	3.71	1.64	0.78	1.01	0.13
C5	S267E/L328F	4.40	1.36	0.36	0.18	10.20	1.42	24.59	4.25	0.62	0.10
Fc-LS	M428L/N434S	2.39	0.49	1.30	0.32	2.52	0.18	0.97	0.04	4.46	0.29
Fc-QL	T250Q/M428L	2.47	0.46	0.59	0.08	0.99	0.21	0.95	0.10	2.39	0.82
Fc-YTE	M252Y/S254T/T256E	0.64	0.15	0.27	0.03	0.27	0.06	0.42	0.01	2.06	0.59
C6A-69	T307A/N315D/A330V/E382V/N389T/N434Y	0.58	0.09	0.65	0.13	0.43	0.05	0.55	0.04	3.67	1.06
C6A-69E	P228R/C6A-69	0.46	0.10	0.67	0.17	0.40	0.03	0.55	0.06	4.40	1.23
C6A-78	T256N/A378V/S383N/N434Y	2.22	0.75	1.88	0.31	3.15	0.65	2.46	0.72	4.10	0.88
C6A-78A	T256N/A378V/N434Y	4.02	0.21	2.38	0.34	2.82	0.57	2.62	0.40	4.72	0.92
C6A-78B	P228L/C6A-78A	1.93	0.21	1.73	0.04	2.27	0.30	2.09	0.29	4.29	0.60
C6A-78D	P228R/C6A-78A	1.90	0.01	2.01	0.28	2.19	0.70	1.86	0.05	4.40	0.98
T5A-74	N315D/A330V/N361D/A378V/N434Y	0.49	0.08	1.77	0.46	0.90	0.31	0.74	0.45	3.50	0.48
T5A-74D	P228R/T5A-74	0.50	0.07	2.16	0.56	0.79	0.29	1.11	0.50	3.33	0.86
T5A-74F	P228R/P230S/T5A-74	0.48	0.17	0.74	0.26	0.58	0.43	0.91	0.59	3.73	0.84
C6A-74	V259I/N315D/N434Y	1.16	0.13	1.09	0.13	1.31	0.15	1.18	0.19	2.83	0.72
C6A-74C	P228L/C6A-74	0.95	0.13	0.89	0.06	1.14	0.13	1.34	0.19	3.03	0.78
C6A-74D	P228R/C6A-74	0.87	0.11	1.02	0.10	1.05	0.05	1.30	0.06	3.48	0.88
C6A-60	P230S/N315D/M428L/N434Y	1.05	0.22	0.47	0.08	1.34	0.25	0.88	0.07	3.76	0.82
C6A-66	E294Del/T307P/N434Y	0.55	0.20	0.08	0.03	0.12	0.07	0.36	0.17	4.41	0.98

*Results are expressed as ratio variant/WT. Increased ratios therefore correspond to improved binding on to the receptor as shown by ELISA assay*.

*0–0.7; 0.7–2; 2–10; >10*.

## Discussion

Due to the remarkable role of FcRn in IgG serum persistence, the Fc/FcRn interactions have been comprehensively studied at the molecular level by mutagenesis (13, 53) and crystallography (59) using mice, rat, and human FcRn. The FcRn-binding site is highly conserved among species and comprises residues located at the CH2/CH3 domains interface: residues 252–254, 307, and 309–311 in the CH2 domain, and residues 433–436 in the CH3 domain. Moreover, for the human Fc/FcRn interaction, the pH dependence has been attributed to the histidine residues H310 and H435, involved in salt bridges with acidic FcRn residues. Various human Fc variants with improved FcRn affinity, and conserved pH dependence, were designed using this structural knowledge and resulted in increased serum half-life. For instance, residues 250 and 428, which are close to the CH2/CH3 interface, were targeted by mutagenesis permitting the isolation of the double variant T250Q/M428L (Fc-QL), exhibiting a ~27-fold binding improvement on human FcRn expressed on the cell surface (16, 17). *In silico* analysis was also used to design several variants combining mutations in or close to the FcRn-binding site. Two of these variants, M428L/N434S (IgG-LS, Xtend) and V259I/V308F/M428L (IgG-IFL), showed a ~11- and ~20-fold improved binding to human FcRn by SPR, respectively, resulting in approximately four- to fivefold half-life extension in cynomolgus monkeys and threefold half-life extension in hFcRn-transgenic mice (18). On the other hand, the triple variant M252Y/S254T/T256E (IgG-YTE) was obtained by phage display selection against murine FcRn of rationally designed libraries targeting Fc residues in or close to the FcRn-binding site (residues 251–256, 308–314, 385–389, and 428–436) (15). When introduced in an anti-RSV and an anti-IL6 IgG1, these YTE mutations increased the IgG binding to human FcRn by about 10-fold by SPR, resulting in a nearly fourfold increase in serum half-life in cynomolgus monkeys (23, 60). Importantly, the anti-RSV YTE variant (motavizumab) resulted in up to fourfold longer half-life in healthy humans, confirming for the first time in humans the concept of half-life extension using Fc-engineered antibodies (26). Interestingly, when the crystal structure of the Fc-YTE mutant (residues 236–444) was compared to its WT counterpart, very few structural changes were observed. Therefore, the greatly enhanced interaction between Fc-YTE and human FcRn is likely mediated by local effects at the substitutions sites (61, 62).

Without using this structural knowledge of Fc/FcRn interaction, we previously identified several mutations in the Fc region, which increase FcRn-binding capacity using a fully random mutagenesis approach combined with a pH-dependent selection process by phage display (41). The use of two successive rounds of random mutagenesis and selection directly allowed finding optimized variants, partly because of synergistic effects of the different mutations. The effect of several of these mutations was unpredictable by rational design as the mutated positions were located distantly from the FcRn-binding site, for example, P228 and P230 in the hinge domain and F241, V264, and A330 in the CH2 domain, and have not been previously described. Nevertheless, the best variants isolated included at least one mutation in the FcRn-binding site (mostly N434S or N434Y). In this study, we used data obtained from random mutagenesis, and from new variants constructed by directed mutagenesis, to verify whether each mutation included in our best variants has indeed a positive effect on the FcRn-binding capacity. Indeed, a minor disadvantage of random mutagenesis is that dispensable mutations could be simultaneously selected and need to be identified afterwards. For one variant only (C6A-78), we identified one mutation that had no effect on FcRn binding (N383S). This result indicates that dispensable mutations are infrequently selected using our system, most of the selected mutations being important for FcRn-binding improvement. Besides, because three single mutations with the greatest FcRn binding were unexpectedly located in the hinge domain (P228L, P228R, and P230S), we cumulated them with our best variants in an attempt to further increase FcRn binding. Surprisingly, additive or neutral effects were observed depending on the parental variant these mutations were added to. These results suggest that designing optimized variants by directed mutagenesis is not straightforward as unpredictable results can be obtained when combining several positive mutations. However, several such rationally designed variants showed further increased FcRn binding by phage-ELISA. When two of these variants were produced as full IgG molecules, C6A-78B (C6A-78/P228L) and C6A-78D (C6A-78/P288R), only the first molecule displayed a modest affinity increase by SPR. This discrepancy could be due to the use of different techniques to measure FcRn-binding. SPR on purified IgG molecules is certainly the most accurate technique but could be less discriminant than the phage-ELISA technique. FcRn-binding improvement up to 28-fold was obtained by phage-ELISA whereas by SPR the best variant showed a 7.4-fold binding increase (41). Alternatively, the discrepancy observed could arise from the phage display system itself. In this system, the Fc fragment is fused to the N-terminal part of the M13 bacteriophage pIII coat protein. Hence, the N-terminal part of the Fc fragment (226–447) is exposed at the phage surface. The position P228 could be less constrained than in the IgG molecule, favoring the replacement by the arginine, while the substitution by the leucine could conserve a positive impact in both systems.

Despite the potential limitations of the phage display system, we and others have clearly shown the advantages of using this system for the selection of engineered Fcs with modulated binding to FcRn (15). In this system, the Fc fragment is aglycosylated but the Fc/FcRn interaction is not dependent on the Fc glycosylation site occupancy (2). Most importantly, the use of the phage display system enables the use of an *in vitro* selection procedure based on the pH dependency of the Fc/FcRn interaction, a specific feature that is critical to preserve. Indeed, increased affinity at high pH could be problematic by preventing the release of FcRn-bound IgGs. Augmented clearance was consequently observed for IgG engineered for improved FcRn affinity at both pH 6.0 and pH 7.4, where pH dependence is reduced (63). Furthermore, recent studies emphasized the difficulty of conserving the pH dependency because when a variant’s affinity to FcRn is increased at pH 6.0, its binding at higher pH increases accordingly (21, 64). Several mutations demonstrating a slight increase in FcRn binding at pH 7.4 were described, among which M252Y and N434Y. Yet, these mutations are included in variants with increased serum half-life (IgG-YTE and our Fc variants, respectively), suggesting that combinations of several mutations could result in different pH dependency or that this parameter is not sufficient to predict half-life modulation. Owing to the difficultly of assessing and interpreting the pH dependence, IgG variants with increased FcRn binding are usually assayed for half-life extension in cynomolgus monkeys and/or in hFcRn mice. These FcRn-humanized mice, unlike wild-type mice, have been shown to be a reliable surrogate for studying human IgG serum half-life (20, 65, 66). Indeed, human and murine FcRn have significant molecular differences, especially concerning pH dependency, rendering wild-type mice an inadequate model for studying half-life of engineered human IgGs (67–70). In these hFcRn mice, several Fc-engineered variants showed equivalent half-life increase (~3-fold), despite distinct affinity improvements, suggesting that Fc engineering may have reached a plateau for FcRn improvement designed to increase half-life (18, 20). Likewise, our best Fc variants administered to hFcRn showed extended half-life, ranging from 1.8- to 2.8-fold increase (41). Most of the variants described herein were not tested in hFcRn mice and we cannot predict their behavior *in vivo*.

A great advantage of the fully random mutagenesis approach used is that identified mutations are distributed all over the length of the Fc region, potentially having an impact on all effector functions. We indeed showed that these FcRn optimized Fc variants display diverse binding capacities to the complement C1q and the FcγRs, resulting in modulated effector activities with increased, conserved, or decreased ADCC and/or CDC activities. Because these activities are essential for the therapeutic efficacy of mAbs, our studies were not limited to one but several Fc variants, which were stored and documented in a database, providing us with distinct “fit-for-purpose” Fc molecules. This database now includes more than 1500 molecules with diverse binding properties. The optimal Fc properties of a given therapeutic mAb are adjustable and will depend on the antibody mode of action (agonistic, cytotoxic, blocking…), and on the antigen and pathology targeted. MAbs can trigger a variety of effects by engaging the complement C1q and different FcγRs expressed by immune cells thus the adequate binding profile for a given molecule needs to be defined. Besides, ADCC activity has been clearly associated with the therapeutic efficacy of mAbs in oncology, but many questions remain concerning the respective roles of ADCC, CDC, and ADCP activities. For other therapeutic applications, like for instance, for anti-viral antibodies, the importance of Fc-dependent effector functions was established for mAbs against several infectious pathogens including HIV (71), RSV (72), and Ebola virus (73). Therefore, many protein-Fc-engineered mAbs with increased binding to FcγRIIIA and/or FcγRIIA (37, 38, 48) but also to the complement C1q (47, 74) have been designed in an attempt to enhance therapeutic efficacy and several such engineered mAbs are now in clinical trials (75, 76). Besides, glyco-engineered mAbs with enhanced ADCC due to low or afucosylation are now used clinically (40). We previously showed that such a glyco-engineering technology, LFB’s EMABling^®^ platform, can be combined with protein-Fc engineering to increase half-life (41). Indeed, when produced in YB2/0 cells, permitting low-fucose content in the Fc glycan moiety, most of our IgG variants conserved improved ADCC, in correlation with FcγRIIIA binding. In contrast, when produced in HEK293 cells, a cell line classically resulting in high fucose content, FcγRIIIA binding results were significantly different from the ADCC results previously obtained. These results suggest that glyco- and Fc-protein engineering can elicit non-compatible effects, resulting in different ADCC activities depending on the production cell line. This divergence between cell lines was not observed when CDC activities and complement C1q binding were compared. This was expected as fucosylation, which is known to have no influence on this effector property.

Finally, the C6A-66 variant showed poor binding to the complement C1q and all FcγRs as well as loss of ADCC and CDC activities. These characteristics are probably due to an unusual deletion of one amino acid, located in position 294. Nonetheless, this variant could be of great interest as for certain clinical applications no effector function could be preferable and, for this variant, ADCC and CDC are completely abolished while half-life is largely enhanced (77). For example, many anti-cytokine mAbs are currently developed as IgG4 or IgG2 mAbs because they display lower effector functions (1, 78, 79), such mAbs could be developed with our C6A-66 variant. The IgG-YTE positive control showed similarly reduced effector activities and decreased binding to the complement C1q and to all FcγRs. The reduced ADCC activity of this variant has been observed before and was restored by addition of mutations S239D/A330L/I332E, known to strongly enhance FcγRIIIA binding (60). We show here that producing the IgG-YTE variant as a low fucosylated molecule cannot restore ADCC activity. Overall, the results obtained for the YTE variant are surprising since this variant comprises three mutations M252Y/S254T/T256E located exclusively in the FcRn-binding site, as clearly observed by crystallography (61). It is noteworthy that C1q and the FcγRs bind to a region involving the hinge and the hinge-proximal portion of the CH2 domain. This region is distant from the CH2/CH3 domain interface containing the residues implicated in FcRn binding. Since mutations at the FcRn-binding site seem to have a great impact on FcγRs and C1q binding as well, these binding sites cannot be considered as independent just because of their relative distant localization. We therefore provide evidence that half-life and effector functions should be optimized simultaneously. Comprehensive understanding of these interactions will not only allow for development of effective therapeutics but also avoidance of potential adverse effects.

## Conflict of Interest Statement

Céline Monnet, Sylvie Jorieux, Nathalie Fournier, Christophe De Romeuf, Rémi Urbain, Christian K. Behrens, Philippe Mondon, and Alexandre Fontayne are employees of LFB Biotechnologies and Khalil Bouayadi was employee of MilleGen, both companies financially supported the study. Results of this study are described in patents WO2010106180 and WO2012175751.

## Supplementary Material

The Supplementary Material for this article can be found online at http://www.frontiersin.org/Journal/10.3389/fimmu.2015.00039/abstract

## Abbreviations

ADCC, antibody-dependent cell-mediated cytotoxicity; ADCP, antibody-dependent cell-mediated phagocytosis; CDC, complement-dependent cytotoxicity; FcγR, Fc gamma receptor; FcRn, neonatal Fc receptor; IgG, immunoglobulin of the G isotype; mAb, monoclonal antibody; NK, natural killer; PK, pharmacokinetics; SPR, surface plasmon resonance; WT, wild-type.
