# Supplementary material for: Selection of IgG Variants with Increased FcRn Binding Using Random and Directed Mutagenesis: Impact on Effector Functions
Source: Front Immunol. 2015 Feb 4;6:39. doi: 10.3389/fimmu.2015.00039 (PMC4316771; doi:10.3389/fimmu.2015.00039)
Supplement: Supplementary file 1 [file DataSheet_1.PDF]

Appendix 1: Amino acid sequence of the Fc fragment of human IgG1 (G1m1,17), residues 216-447 corresponding to the hinge, CH2 and CH3 domains.

216 ***hinge*** 231 250 CH2 270

EPKSCDKTHTCPPCPAPELLGGPSVFLFPPKPKDTLMISRTPEVTCVVVDVSHEDPEVKFNW

290 CH2 310 330

YVDGVEVHNAKTKPREEQYNSTYRVVSVLTVLHQDWLNGKEYKCKVSNKALPAPIEKTISKA

341 360 **CH3** 380

K**GQPREPQVYTLPPSRDELTKNQVSLTCLVKGFYPSDIAVEWESNGQPENNYKTTPPVLD**

400 **CH3** 420 440

SDGSFFLYSKLTVDKSRWQQGNVFSCSVMHEALHNHYTQKSLSLSPGK
